# The Effect of Social Support on Recovery and Treatment Adherence in Individuals Diagnosed with Bipolar Disorder

**DOI:** 10.1192/j.eurpsy.2024.913

**Published:** 2024-08-27

**Authors:** R. Aydar, M. C. Aktaş, C. H. Ayhan

**Affiliations:** Van Yüzüncü Yıl University, Van, Türkiye

## Abstract

**Introduction:**

Social support have affected the recovery and adherence with treatment in bipolar patients.

**Objectives:**

This research aims to understand the contribution of social support mechanisms to treatment adaptation processes in individuals struggling with bipolar disorder and the effects of these support mechanisms on recovery. In this way, the importance of the social support factor will be tried to be understood in order to provide more effective and customized support to individuals living with bipolar disorder.

**Methods:**

This study will planned to descriptive correlational design. The data will collect to using the Morisky Treatment Adherence Scale (MTAS), the Multidimensional Scale of Perceived Social Support (MSPSS), the Recovery Process Inventory (RPIS), and the Sociodemographic Data Form from individuals diagnosed with Bipolar disorders. By filling out these scales, participants will evaluate their treatment compliance, perceived social support levels, and recovery processes. The data will be subjected to appropriate methods for statistical analysis and will be used to understand the relationships between social support and treatment compliance and recovery processes.

**Results:**

Data extraction is still on going in detailed style by principal authors. Description of studies and the key findings will be presented.

**Conclusions:**

It is thought that the results obtained from this research will be an important guide in providing more effective support to individuals with the level of social support, treatment adherence and recovery processes in individuals struggling with bipolar disorder

**Key Words:**

bipolar disorder, social support, treatment adherence, recovery.

**Disclosure of Interest:**

None Declared

